# Transcriptome Sequencing Reveals That Intact Expression of the Chicken Endogenous Retrovirus *chERV3* In Vitro Can Possibly Block the Key Innate Immune Pathway

**DOI:** 10.3390/ani13172720

**Published:** 2023-08-26

**Authors:** Xi Zhang, Tingting Xie, Xiaoqi Li, Min Feng, Guodong Mo, Qihong Zhang, Xiquan Zhang

**Affiliations:** 1 Guangdong Provincial Key Laboratory of Agro-Animal Genomics and Molecular Breeding, College of Animal Science, South China Agricultural University, Guangzhou 510642, China; zhangxi052627@163.com (X.Z.);; 2 Key Laboratory of Chicken Genetics, Breeding and Reproduction, Ministry of Agriculture, Guangzhou 510642, China

**Keywords:** chicken, endogenous retroviruses, innate immune, RNA-seq, Toll-like receptor signaling pathway

## Abstract

**Simple Summary:**

Endogenous retroviruses (ERVs) are ancient viral sequences that have integrated into the genomes of vertebrates. In order to evaluate the function of *chERV3* on the host innate immune response, a full-length (10,218 bp) reverse cloning plasmid (puc57-chERV3) was constructed and transfected into primary chicken embryo fibroblasts (CEFs) for transcriptome sequencing. We then performed transcriptome sequencing to analyze the gene expression changes induced by *chERV3*. We found that *chERV3* down-regulated many genes involved in immune-related processes, such as the inflammatory response, innate immune response, and the Toll-like receptor signaling pathway. These processes are involved in detecting and responding to viral infection. Our study systematically identified the effect of *chERV3* prokaryotic expression on the innate immune response in chickens. ERVs are a rich source of genetic diversity and innovation in vertebrates, and understanding their roles and mechanisms may have implications for susceptibility to animal diseases.

**Abstract:**

Endogenous retroviruses (ERVs) are viral sequences that have integrated into the genomes of vertebrates. Our preliminary transcriptome sequencing analysis revealed that *chERV3* is active and is located on chromosome 1:32602284–32615631. We hypothesized that *chERV3* may have a role in the host innate immune response to viral infection. In this study, using reverse genetics, we constructed the puc57-chERV3 full-length reverse cloning plasmid in vitro. We measured the p27 content in culture supernatant by enzyme-linked immunosorbent assay (ELISA). Finally, transcriptome analysis was performed to analyze the function of *chERV3* in innate immunity. The results showed that *chERV3* may generate p27 viral particles. We found that compared to the negative control (NC) group (transfected with pMD18T-EGFP), the *chERV3* group exhibited 2538 up-regulated differentially expressed genes (DEGs) and 1828 down-regulated DEGs at 24 hours (h) and 1752 up-regulated DEGs and 1282 down-regulated DEGs at 48 h. Based on Gene Ontology (GO) and Kyoto Encyclopedia of Genes and Genomes (KEGG) pathway enrichment analyses, the down-regulated DEGs were enriched mainly in immune-related processes such as the inflammatory response, innate immune response, and Toll-like receptor signaling pathway. GSEA showed that the Toll-like receptor signaling pathway was suppressed by *chERV3* at both time points. We hypothesized that *chERV3* can influence the activation of the innate immune pathway by blocking the Toll-like receptor signaling pathway to achieve immune evasion.

## 1. Introduction

Retroviruses are viruses that can insert their genetic material into the genomes of their hosts. Sometimes, this happens in germ cells. When this happens, the retroviral sequences become part of the host’s hereditary DNA and are passed on to future generations; these sequences are called endogenous retroviruses (ERVs) [1]. ERVs accumulate many mutations as the host develops, and many ERVs are inactivated as a way to prevent ERVs from assembling viral particles that can cause harm to the host. However, as modern sequencing technologies continue to mature, an increasing number of ERVs are found to still have intact sequences and the ability to function; one study identified 974 different ERVs by whole-genome sequencing of 407 local breeds of chickens with obERVer [2]. In chickens, there are four main groups of ERVs: ALVE, ERV, CR1, and ART-CH [3].

ERVs can affect various aspects pertaining to host physiology and disease, such as placental development [4], cancer [5], reproduction [6], and autoimmune diseases [7]. ERVs can interact with the host’s innate immune system, which is the first line of defense against viral infection [8]. The innate immune system can recognize ERVs as foreign invaders by detecting their double-stranded RNA (dsRNA) molecules. These molecules activate receptors such as Toll-like receptors (TLRs) and RIG-I-like receptors (RLRs), triggering the production of type I interferon and other antiviral factors [9,10]. However, the expression of ERVs can be suppressed by immune factors such as interferon regulation factor (IRF) [11] and lysine acetyltransferase TP60 [12]. When these factors are removed, ERVs can reactivate and induce inflammatory responses mediated by STING and IRF7 [12]. These interactions between ERVs and the innate immune system may have implications for the host’s susceptibility or resistance to viral infection.

We preliminarily identified ERVs genome-wide in chicken (Gallus_gallus 5.0) and then named the 436 newly identified ERVs according to their positions on chromosomes. The qPCR results showed that *chERV3*, located at chromosome 1 position 32603303–32610827, was able to affect the expression of some interferon-stimulated genes (ISGs) [13]. We hypothesized that *chERV3* is able to affect the immune response, but few genes were detected in our preliminary analysis, and the effect of *chERV3* prokaryotic expression on the activation of the innate immune response in chickens is still unclear. In this study, we used RNA-seq to study the changes in the innate immune response in chickens after prokaryotic expression of *chERV3* preliminarily assess the effect of ERVs on the innate immune responses of birds and to accumulate data to support breeding for resistance to avian leukemia.

## 2. Materials and Methods

### 2.1. Construction of the chERV3 Full-Length Reverse Cloning Vector

To synthesize full-length *chERV3* (7524 bp), we used a fragment assembly strategy. We first split the full-length sequence into six large fragments and then further divided each large fragment into 5–7 small fragments. We designed primers for each small fragment and synthesized them. Then, we assembled the small fragments into the corresponding large fragments. Finally, we joined the six large fragments together to obtain the full-length *chERV3* sequence and cloned the full-length sequence into the puc57 prokaryotic expression vector. The primer information is provided in Supplementary File S1.

### 2.2. Cells and Transfection

Chicken embryo fibroblast (CEF) cells were isolated from trunk tissues of 9- to 11-day-old SPF eggs (Xinxing Dahuanong Poultry Egg, Guangzhou, China). The CEFs were cultured in Dulbecco’s modified Eagle’s medium (DMEM) (Gibco, Grand Island, NY, USA) supplemented with 5% FBS and incubated at 37 °C with 5% CO_2_. CEFs were transfected with the puc57-*chERV3* plasmid using Lipofectamine 3000 reagent (Thermo Fisher, Waltham, MA, USA) following the manufacturer’s protocol.

### 2.3. ELISA

An ELISA was used for the detection of p27 levels in the supernatant and pellet of puc57-chERV3 plasmid-transfected CEFs. The transfected CEFs were subjected to three cycles of freeze-thaw. The supernatant and the cell pellet were collected separately. The supernatant was assayed for p27 using the avian leucosis virus antigen test kit (IDEXX, Westbrook, ME, USA). The viremia results were expressed as S/P ratios (relative antigen content). An S/P ratio higher than 0.2 indicated a positive result, and an S/P ratio lower than 0.2 indicated a negative result.

### 2.4. Sample Collection

Total RNA for RNA-seq was isolated from CEFs transfected with the puc57-chERV3 plasmid at 24 and 48 h using TRIzol reagent (Invitrogen, Carlsbad, CA, USA). Samples were collected from three independent experiments. CEFs transfected with the puc57-EGFP plasmid were used as the control group. The purity and quantity of the total RNA were assessed using a NanoPhotometer spectrophotometer (Implen, Westlake Village, CA, USA) and an Agilent 2000 (Agilent, Santa Clara, CA, USA). RNA degradation and contamination were further monitored using agarose gel electrophoresis.

### 2.5. Library Preparation for Transcriptome Sequencing

Total RNA was used as input material for the RNA sample preparations. Briefly, mRNA was purified from total RNA using poly-T oligo-attached magnetic beads. Fragmentation was carried out using divalent cations under elevated temperature in First-Strand Synthesis Reaction Buffer (5×). First-strand cDNA was synthesized using a random hexamer primer and M-MuLV Reverse Transcriptase (RNase H^−^). Second-strand cDNA synthesis was subsequently performed using DNA Polymerase I and RNase H. Remaining overhangs were converted into blunt ends via exonuclease/polymerase activities. After the adenylation of 3′ ends of DNA fragments, an adaptor with hairpin loop structure was ligated to prepare for hybridization. In order to select cDNA fragments of preferentially 370~420 bp in length, the library fragments were purified with AMPure XP system (Beckman Coulter, Brea, CA, USA). At last, PCR products were purified (AMPure XP system), and library quality was assessed on the Agilent Bioanalyzer 2100 system. At last, the library preparations were sequenced on an Illumina Novaseq platform 6000.

### 2.6. Quality Control and Mapping

Raw data (raw reads) of fastq format were firstly processed through fastp software (CASAVA 1.8.2). In this step, clean data (clean reads) were obtained by removing reads containing the adapter, reads containing 1 ploy-N, and low-quality reads from raw data. At the same time, Q20, Q30, and GC content and the clean data were calculated. All the downstream analyses were based on the clean data with high quality. Index of the reference genome (Gallus_gallus 6.0) was built using Hisat2 v2.0.5, and paired-end clean reads were aligned to the reference genome using Hisat2 v2.0.5.

### 2.7. Quantification of Gene Expression Level

Feature Counts (v1.5.0) was used to count the reads numbers mapped to each gene. And then, the FPKM of each gene was calculated based on the length of the gene and reads count mapped to this gene.

### 2.8. Differential Expression Analysis

Differential expression analysis of two conditions/groups (two biological replicates per condition) was performed using the DESeq2 R package (1.20.0). The resulting *p*-value were adjusted using the Benjamini and Hochberg’s approach for controlling the false discovery rate. Genes with an adjusted *p* value < 0.05 found by DESeq2 were assigned as differentially expressed.

### 2.9. GO and KEGG Enrichment Analysis of Differentially Expressed Genes

GO enrichment analysis and KEGG analysis of differentially expressed genes were implemented by the clusterProfiler R package (3.8.1). GO terms and KEGG pathway with corrected *p*-value < 0.05 were considered significantly enriched by differential expressed genes.

### 2.10. Gene Set Enrichment Analysis (GSEA)

We use the local version of the GSEA analysis tool (http://www.gsea-msigdb.org/gsea, accessed on 13 January 2023), GO and KEGG dataset were used for GSEA independently.

### 2.11. Validation of DEGs Using qPCR

For gene expression analysis, cDNA synthesis was performed using an All-in-one RT SuperMix Perfect for qPCR kit (Vazyme, NanKing, China) according to the manufacturer’s instructions. qPCR was used to verify the levels of expressed genes, with glyceraldehyde-3-phosphate (GAPDH) used as the internal reference gene. Vazyme ChamQ Universal SYBR qPCR Master Mix Q711-02 (Vazyme, NanKing, China) and Bio-Rad CFX96 (Bio-Rad, Hercules, CA, USA) were used for qPCR. Information on the qPCR primers is shown in Supplementary File S2.

### 2.12. Statistical Analysis

Statistical comparisons were performed using GraphPad Prism 9 (GraphPad Software Inc., San Diego, CA, USA). All statistical data are presented as the mean ± SEM. Gene expression was calculated using the relative quantification (2^−ΔΔct^) method. Statistical significance is indicated by the *p*-value: >0.05 (nonsignificant, ns), <0.05 (*), 0.01 (**), or 0.001 (***).

### 2.13. Raw Data Information

The data obtained via RNA-seq were deposited into the NCBI database under accession number PRJNA967101.

## 3. Results

### 3.1. Construction of the chERV3 Full-Length Reverse Cloning Vector

**Figure 1:**
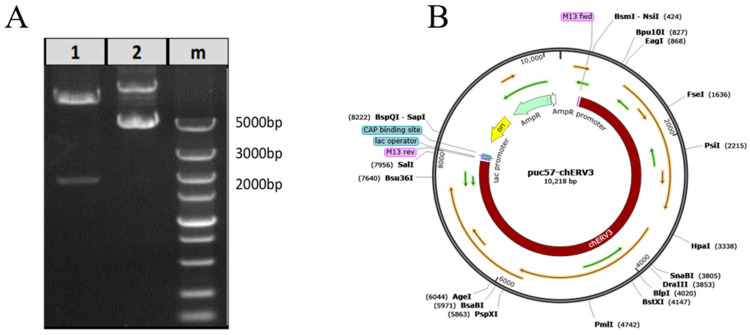
Construction of the full-length reverse cloning plasmid of puc57-chERV3. Note: (**A**) digestion analysis of the puc57-chERV3 plasmid by *Eco*RV (lane 1); (**B**) full-length sequence of the puc57-chERV3 plasmid.

We constructed the puc57-chERV3 full-length reverse cloning plasmid in vitro and then validated the plasmid by digestion with the *Eco*RV enzyme. The results showed that we obtained the correct fragment (Figure 1A). The plasmid structure is shown in Figure 1B.

### 3.2. chERV3 Is Able to Release p27 Antigen

**Figure 2:**
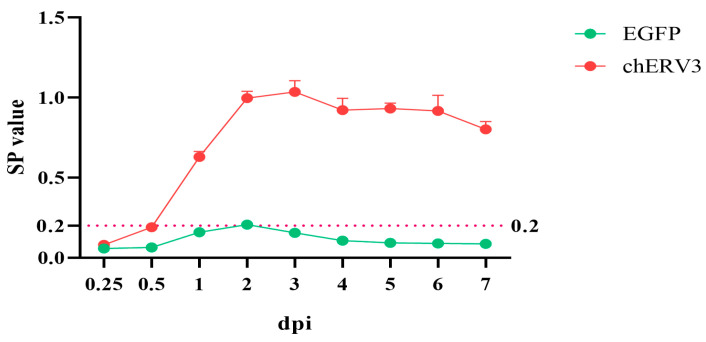
The *chERV3* viral capsid protein (p27) level was measured by ELISA. The Y-axis is the value of SP, and the X-axis is the time of *chERV3* transfection in CEFs.

We transfected CEFs with the puc57-chERV3 plasmid and collected supernatant with cell freeze–thaw samples 6 h, 12 h, and 1–7 d after *chERV3* transfection. We performed an ELISA assay to detect the p27 level in CEFs and expressed it as the S/P ratio. The results revealed that no p27 antigen was detected at 6 h or 12 h post transfection, but p27 antigen was detected after 24 h, and the level increased gradually until 72 h, when it peaked. The p27 level then declined slightly from 96 h to 168 h (Figure 2). The results imply that *chERV3* is a functionally normal ERV. The SP value data are shown in Supplementary File S3.

### 3.3. Sequence Data Quality Statistic

We sequenced the transcriptomes of CEFs transfected with puc57-chERV3 or pMD18T-EGFP (NC) at 24 h and 48 h after transfection. As Table 1 demonstrates, we performed quality control on the sample sequencing data. The integrity of each RNA sample surpassed QC standards. Each sample had clean bases totaling over 5.9 G data, resulting in a total data volume of over 70 G from 12 samples. We aligned the sequencing data with the whole chicken genome (Gallus_gallus 6.0). The base identification correct rate was above 96%, and the comparison rate for each sample surpassed 85%. Thus, the sequencing data were of high quality and suitable for bioinformatics analysis. We also calculated the FPKM value of *chERV3* (including a part of the sequence around *chERV3*) in each sample and found that it was higher in the puc57-chERV3 group than in the NC group (Supplementary File S4).

### 3.4. Differential Gene Expression Identification after puc57-chERV3 Transfection in CEFs

**Figure 3:**
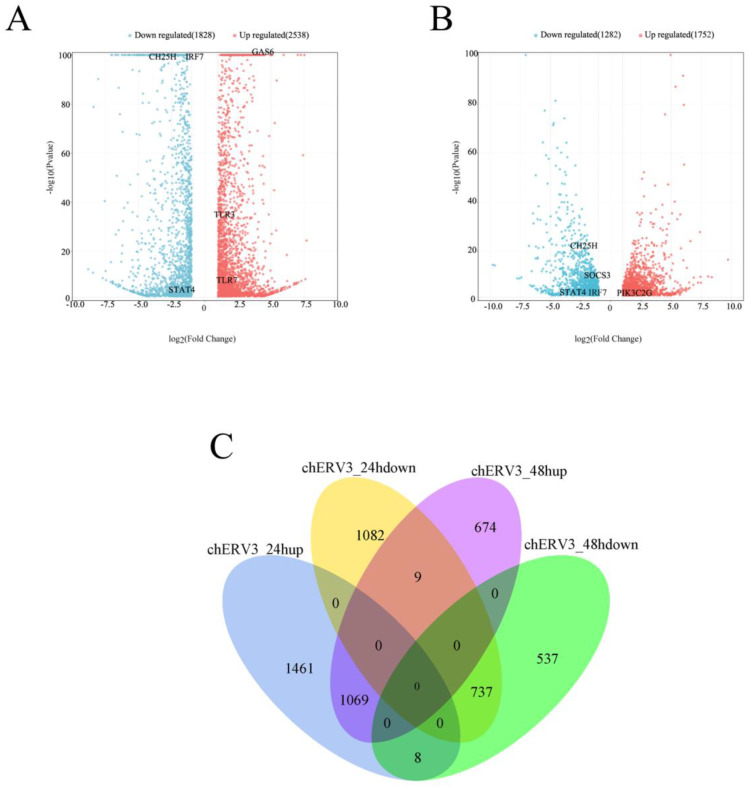
DEGs in CEFs transfected with *chERV3* at 24 and 48 h. Note: (**A**) statistical chart of all DEGs in CEFs transfected with *chERV3* at 24 h; (**B**) statistical chart of all DEGs in CEFs transfected with *chERV3* at 48 h. The Y-axis is the value of −log_10_ (*p* value), and the X-axis is the value of log_2_ (Fold Change); (**C**) Venn diagrams of up-regulated and down-regulated DEGs in CEFs transfected with *chERV3* at 24 and 48 h.

We identified the differentially expressed genes (DEGs) between the puc57-chERV3 group and the NC group at 24 and 48 h after transfection. We used a cutoff of |log2FC| > 1 and FDR < 0.05 to define significant DEGs. We found that at 24 h, there were 2538 up-regulated DEGs and 1828 down-regulated DEGs in the puc57-chERV3 group compared to the NC group (Figure 3A). At 48 h, there were 1752 up-regulated DEGs and 1282 down-regulated DEGs in the puc57-chERV3 group compared to the NC group (Figure 3B). We screened some immune-related genes, as shown in Figure 3A,B.

We also compared the DEGs at the two time points and found that there were some unique DEGs at each time point. At 24 h, there were 1461 up-regulated DEGs and 1082 down-regulated DEGs that were not present at 48 h. At 48 h, there were 674 up-regulated DEGs and 537 down-regulated DEGs that were not present at 24 h. There were 1069 up-regulated and 737 down-regulated DEGs in common at each of the two time points (Figure 3C).

We also screened some of the innate immune-related genes among the DEGs. These genes included pattern recognition receptors (*TLR1*, *TLR4*, and *TLR7*), interferon genes (*IFN-α*, *IFN-β*), interferon regulatory factor (*IRF7*), interferon-stimulated genes (*CH25H* and *STAT4*), and inflammatory factors (*IL-1*, *IL-6*, and *IL-12*). Most of these genes were down-regulated by *chERV3* at both time points.

In addition, we identified some DEGs that had opposite expression patterns at 24 and 48 h. There were eight DEGs (*SCEL*, *MYCBP2*, *LMO7*, *PCDH9,* and two new transcripts) that were up-regulated at 24 h but down-regulated at 48 h. There were nine DEGs (*PIK3C2G*, *SFRP4*, *NDST4*, *P3H2*, *MXD3*, *SLC25A48*, *APOA1*, *DIXDC1,* and a new transcript) that were down-regulated at 24 h but up-regulated at 48 h. Information on the DEGs is shown in Supplementary File S5.

### 3.5. GO Annotation of DEGs after puc57-chERV3 Transfection in CEFs

**Figure 4:**
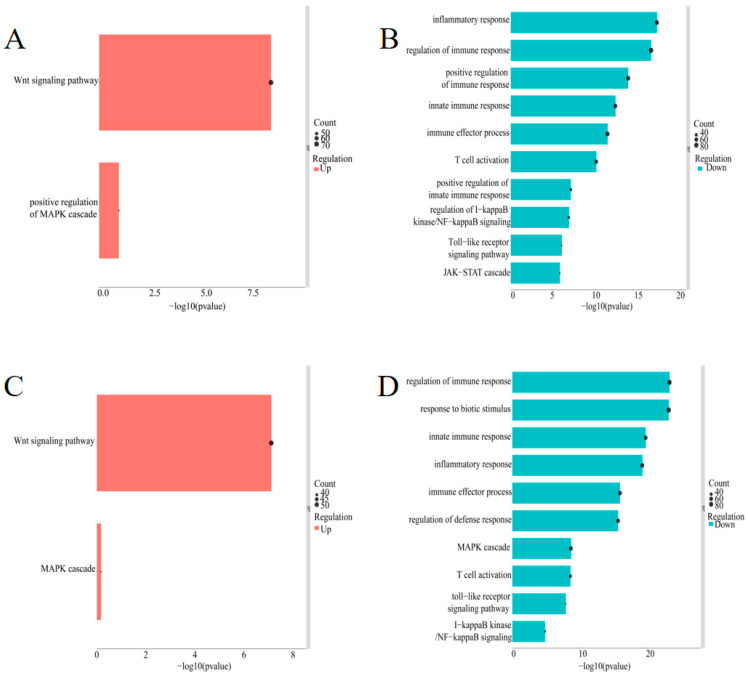
GO term analysis of down-regulated DEGs in CEFs transfected with *chERV3* at 24 and 48 h; (**A**) up-regulated DEGs at 24 h; (**B**) down-regulated DEGs at 24 h; (**C**) up-regulated DEGs at 48 h; and (**D**) down-regulated DEGs at 48 h.

We performed GO biological process analysis to annotate the functions of the DEGs. We found that the up-regulated DEGs were mainly enriched for the Wnt signaling term (Figure 4A,C), which is involved in cell proliferation and differentiation. The down-regulated DEGs induced by *chERV3* at 24 h were enriched for processes such as the inflammatory response, innate immune response, T-cell activation, Toll-like receptor signaling pathway, NF-κB signaling pathway, and JAK-STAT signaling pathway (Figure 4B). The down-regulated DEGs induced by *chERV3* at 48 h were enriched for processes such as biotic stimulus response, innate immune response, inflammatory response, MAPK pathway, T-cell activation, Toll-like receptor signaling pathway, and NF-κB signaling pathway (Figure 4D). At both time points, *chERV3*-induced downregulation of DEGs was significantly enriched in the inflammatory response, innate immune response, and TLR signaling terms. Some of the immune-related genes were *IL-6*, *IL-1*, *IFN-β*, *IFN-α*, *TNF-α*, *TRAF3*, *NF-κB*, *IRF1*, *IRF7*, *PI3KCA*, and others. More details of the DEGs involved in GO enrichment analysis can be found in Supplementary File S6.

### 3.6. RNA-seq Data Matched the qPCR Data

**Figure 5:**
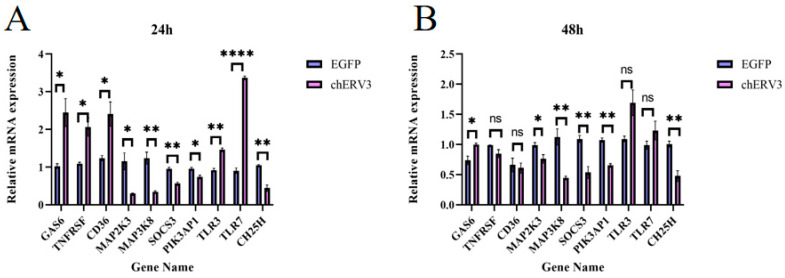
Validation of RNA-seq data by qPCR. (**A**) *chERV3* transfected in CEFs at 24 h, (**B**) *chERV3* transfected in CEFs at 48 h. Blue represents NC, and purple represents *chERV3* (* *p* < 0.05; ** *p* < 0.01; **** *p* < 0.0001; ns: Non-significant).

Using gene function annotation, we screened 10 genes related to innate immunity, including some interferon-stimulated genes (ISGs) such as *GAS6*, *TLR3*, *TNFRSF19*, *TLR7*, *CD36*, *MAP2K3*, *MAP3K8*, *SOCS3*, *PIK3AP1*, and *CH25H* (Table 2). To validate the RNA-seq results, we performed qPCR to verify the expression of the 10 DEGs in Table 2 The *P* values of each gene are listed in the Supplementary Table S1. The qPCR results were consistent with the sequencing results. *GAS6* expression was significantly up-regulated at 24 and 48 h after *chERV3* transfection in CEFs (Figure 5A,B), while *SOCS3*, *CH25H,* and *PIK3AP1* were significantly down-regulated at 24 and 48 h after *chERV3* transfection (Figure 5A,B).

### 3.7. Pathway Analysis of DEGs after puc57-chERV3 Transfected in CEF Cells

**Figure 6:**
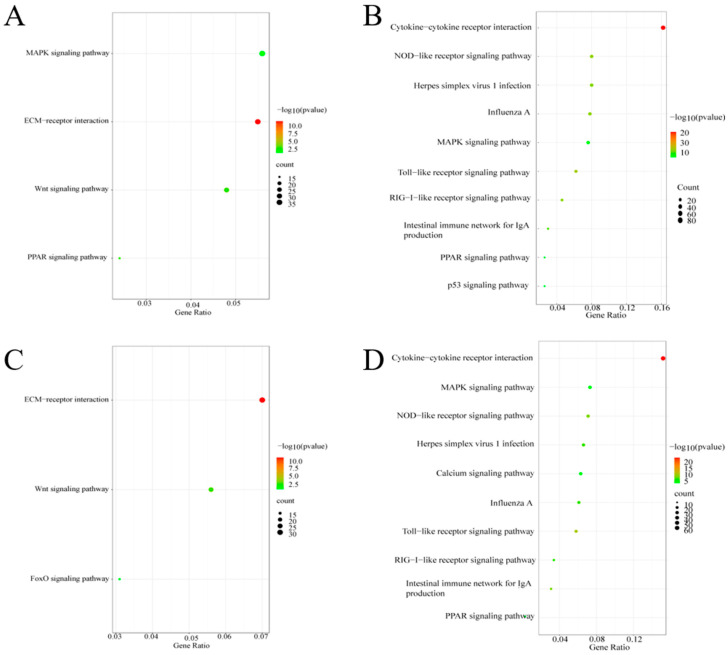
KEGG pathways of up-regulated and down-regulated DEGs expressed in CEFs transfected with *chERV3* at 24 and 48 h; (**A**) up-regulated DEGs at 24 h; (**B**) down-regulated DEGS at 24 h; (**C**) up-regulated DEGS at 48 h; and (**D**) down-regulated DEGs at 48 h.

We performed KEGG pathway analysis to annotate the functions of the DEGs. We found that the DEGs were involved in 156 KEGG pathways at 24 h and 153 KEGG pathways at 48 h. The up-regulated DEGs were mainly enriched for pathways related to cell proliferation and differentiation, such as ECM receptor interaction, MAPK signaling pathway, Wnt signaling pathway, and PPAR signaling pathway (Figure 6A,C). The down-regulated DEGs were mainly enriched for pathways related to the immune response and inflammation, such as cytokine-cytokine receptor interaction, Toll-like receptor signaling pathway, and RIG-I-like receptor signaling pathway (Figure 6B,D). We observed that the DEGs that were up-regulated by *chERV3* were mainly involved in ECM receptor interactions. However, the DEGs that were down-regulated by *chERV3* were mostly related to the Toll-like receptor signaling pathway. More details of the KEGG pathway analysis can be found in Supplementary File S7.

### 3.8. chERV3 Is Able to Induce Downregulation of the Toll-like Receptor Signaling Pathway

**Figure 7:**
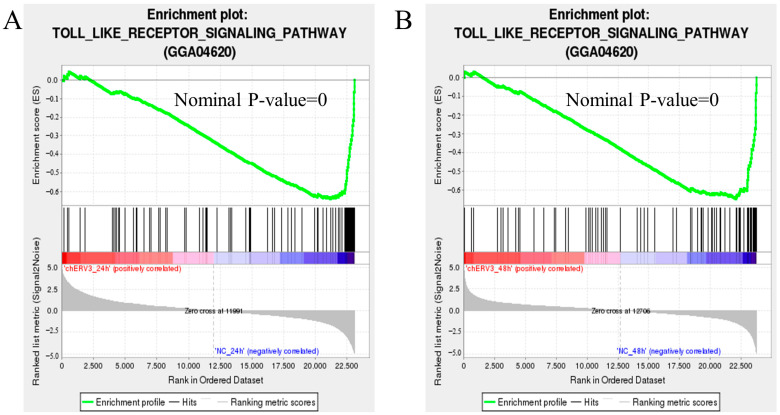
Expression validation of the Toll-like receptor signaling pathway. (**A**,**B**) Gene set enrichment analysis of the Toll-like receptor signaling pathway in CEFs transfected with *chERV3* at 24 h and 48 h.

**Figure 8:**
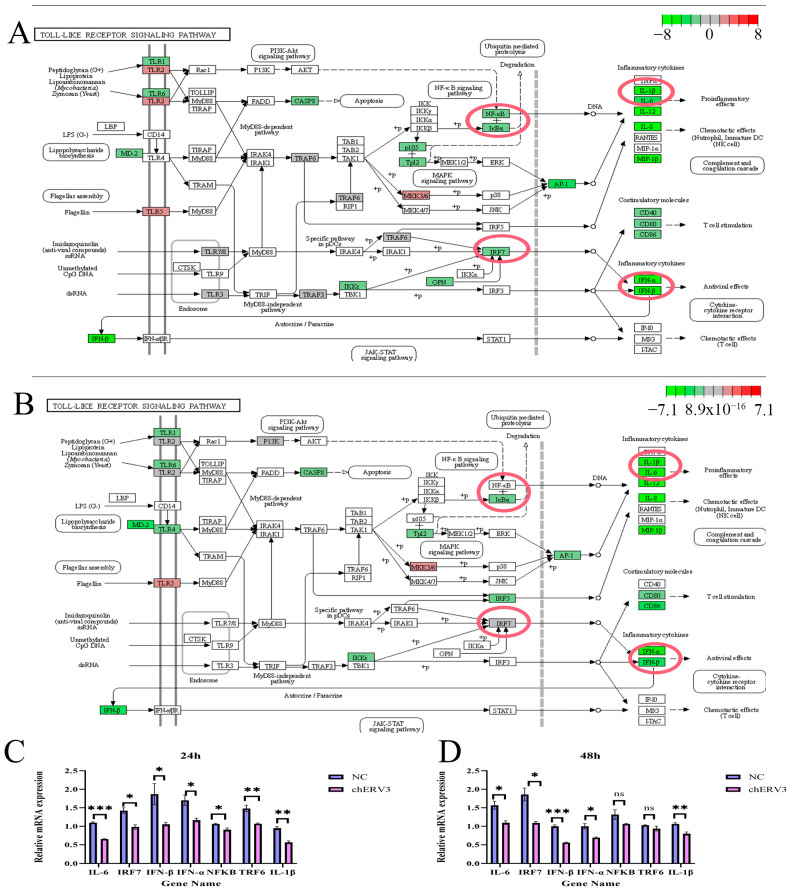
Identification of the expression of key genes of the TLR signaling pathway. Note: (**A**,**B**) Toll-like receptor signaling pathway (24 and 48 h), we have circled seven immune-related genes in red; (**C**,**D**) qPCR verification of DEGs at 24 and 48 h. Blue represents NC and purple represents *chERV3* (* *p* < 0.05; ** *p* < 0.01; *** *p* < 0.001).

In GO and KEGG enrichment analysis, we found that *chERV3* induced the downregulation of DEGs significantly enriched in immune-related processes, for which the Toll-like receptor signaling pathway was screened at 24 and 48 h. GSEA illustrated that *chERV3* significantly down-regulated Toll-like receptor signaling pathway gene expression at 24 and 48 h (Figure 7A,B). We identified seven immune-related DEGs from the Toll-like receptor signaling pathway (KEGG ID: gga04620), including *IL-6*, *IL-1β*, *IRF7*, *IFN-α*, *IFN-β*, *NF-κB*, and upstream genes related to NF-κB: *TRF6* and *IKKα* (Figure 8A,B). The *P* values of each gene are listed in the Supplementary Table S1. The qPCR results were consistent with the RNA-seq results (Figure 8C,D)*. chERV3* induced significant downregulation (*p* < 0.05) of *IL-6* (24 h, 48 h), *IL-1β* (24 h, 48 h), *IRF7* (24, 48 h), *IFN-β* (24, 48 h), *IFN-α* (24, 48 h), *NF-κB* (24 h), and *TRF6* (24 h) expression; at the 48 h time point, *chERV3* induced downregulation of *NF-κB* and *TRF6* expression, albeit not significantly (*p* > 0.05). We speculate that the expression of the Toll-like receptor signaling pathway can be down-regulated by *chERV3*.

## 4. Discussion

Accumulating studies are showing that ERVs can influence host physiological processes, especially the immune response. The expression of *TLR3* is dramatically increased following transfection of *lnc-ALVE1-AS1* in MDMs, and it has been demonstrated that *lnc-ALVE1-AS1* may directly generate a dsRNA structure that *TLR3* can recognize [14,15]. One-third of the binding sites for the tumor suppressor *p53* are adjacent to ERV sequences [16]. Comparatively few investigations have been conducted on the transcriptional expression functions of chicken ERVs themselves. And in our previous study, we found that *chERV3* has a complete structure with long terminal repeats (LTR) on both ends. Generally, LTR consists of 3′ unique region U3, short repeat region R, and 5′ unique region U5, which are mainly responsible for viral replication, translation, RNA processing, and viral integration into the host genome. The 5′LTR contains the promoter and enhancer signals initiating transcription, while the 3′LTR contains the polyadenylation signal terminating transcription [17]. For example, as an exogenous retrovirus, HIV 5′LTR contains both enhancer and promoter elements. In HIV infection, the dual LTR is capable of breaching the genome’s immunity to replicate HIV within intestinal macrophages and CD4 T cells [18]. However, endogenous retroviruses have a functionally weaker LTR region than exogenous retroviruses, but some endogenous LTRs still have strong RNA Polymerase II regulatory sequences [19,20], and contain a plethora of transcription factor binding sites [21]. Endogenous retroviruses’ LTR region can also function as a promoter to control the expression of ERVs [22]. During the long evolutionary process, many endogenous retroviruses excise their internal coding regions by homologous recombination of the LTRs on both sides, becoming a single LTR region [23,24], but can nevertheless still regulate nearby gene expression. It has been shown that the LTR of endogenous retroviruses is able to act as a promoter to regulate the ectopic expression of CYP19A1 encoding an aromatase in the skin [25]. Additionally, it was discovered in research on human endogenous retroviruses that the ERV9-LTR was subsequently found to control the expression of several genes, many of which are involved in immunity or apoptosis [26]. Therefore, we hypothesized that the LTR region of *chERV3* can regulate *chERV3* transcriptional expression; however, there is still much to learn about how *chERV3-LTR* works. Especially, further research is needed to determine whether the LTR of *chERV3* can independently exert immunological functions. Actually, exploring the natural functions of LTR beyond the full-length ERV is a challenge. Comparatively few investigations have been conducted on the transcriptional expression functions of chicken ERVs themselves, however, because of the high homology of ERVs and their unknown genomic locations, time dependency, and methods of expression. In this study, we constructed a full-length reverse cloning plasmid of puc57-chERV3 in vitro. The ELISA results revealed that p27 antigen was not detected at 6 h and 12 h post transfection but was detected after 24 h, and its level increased gradually until peaking at 72 h. These results implied that *chERV3* is a functionally normal ERV.

Then, we used RNA-seq analysis to verify the effect of *chERV3* on the host innate immune response. In CEFs, *chERV3* prokaryotic expression induced the expression of several immune response-related DEGs. Toll-like receptors were screened in this analysis: At 24 h post transfection, *chERV3* prokaryotic expression induced significant upregulation of *TLR3*, *TLR5,* and *TLR7*; and at 48 h, *chERV3* prokaryotic expression induced significant downregulation of *TLR1* and *TLR4* expression. *TLR1*, *TLR3*, *TLR4*, *TLR5*, and *TLR7* all belong to the TLR family, and TLRs are the most typical family of PRRs that recognize PAMPs [27]. In addition, during the analysis of the DEGs induced by *chERV3* prokaryotic expression, we found that *chERV3* induced a significant downregulation of *PIK3C2G* at 24 h and an upregulation of *PIK3C2G* at 48 h. *PIK3C2G* belongs to class II phosphoinositide 3-kinases (PI3Ks) [28]. PI3Ks have been shown to play critical roles in signaling pathways that regulate proliferation, oncogenic transformation, cell survival, cell migration, and intracellular protein trafficking. *PIK3CG* is currently considered a class of tumor suppressor genes [29]. We hypothesized that *chERV3* is recognized by TLRs after prokaryotic expression but is likely to suppress the natural host immune response as the time of *chERV3* prokaryotic expression increases.

Other studies have also shown that ERVs can affect the innate immune response of the host. For instance, some ERVs can block the receptors for exogenous retroviruses or induce immune tolerance to prevent excessive inflammation [30]. Some human ERVs can also produce immunosuppressive RNA, DNA, or proteins that dampen the immune response [31]. Moreover, some chicken breeds that carry ERVs have weaker cellular and humoral immunity and are more prone to viral infection than other breeds that do not carry ERVs [32]. We performed GO enrichment analysis on the DEGs induced by *chERV3*. GO enrichment analysis showed that the DEGs with *chERV3*-induced downregulation were significantly enriched in immune-related processes such as the inflammatory response, immune response, and Toll-like receptor signaling pathway. These processes were suppressed at both 24 and 48 h after *chERV3* prokaryotic expression. Subsequently, we performed clustering analysis of the genes enriched in the immune-related GO process and found that *chERV3*-induced immune-associated DEGs with similar expression patterns were clustered together and that most DEGs showed fluctuations in expression at both time points. We selected 10 of these DEGs (*GAS6*, *TLR3*, *TNFRSF19*, *TLR7*, *CD36*, *MAP2K3*, *MAP3K8*, *SOCS3*, *PIK3AP1*, and *CH25H*) for qPCR validation, and the results were consistent with the RNA-seq results. Among these genes, *GAS6* was significantly up-regulated in response to *chERV3*. *GAS6* interacts with Tyro3, Axl, and Mer (TAM) receptors through its sex-hormone-binding globulin (SHBG) domain and activates downstream signaling, such as the phosphatidylinositol 3-kinase (PI3K), extracellular signal-regulated kinase (ERK), and nuclear factor kappa-light-chain-enhancer of activated B-cells (NF-κB) pathways, to regulate cellular proliferation, migration, differentiation, adhesion, and apoptosis [33,34]. Moreover, *chERV3* induced significant downregulation of *SOCS3* and *CH25H*; *SOCS3* belongs to the suppressor of cytokine signaling (SOCS) family of proteins and binds to Jak-proximal sites on cytokine receptors to inhibit Jak activity [35]. *CH25H* is a classical ISG and can broadly inhibit membrane fusion of enveloped viruses by converting cholesterol to 25-hydroxycholesterol (25HC) [36]. We hypothesized that *chERV3* induces downregulation of DEGs significantly enriched in immune-related pathways; however, the effect of *chERV3* on immune-related DEGs is not singular, and the exact mechanism remains unclear.

Subsequently, we performed KEGG enrichment analysis of *chERV3*-induced DEGs and found that at both 24 and 48 h, *chERV3* prokaryotic expression induced downregulation of DEGs significantly enriched in the Toll-like signaling pathway, NOD-I-like receptor signaling pathway and RIG-I-like receptor signaling pathway. Further, immune-related down-regulated DEGs, including *IL-6*, *IL-1β*, *IFN-β*, *IFN-α*, *TNF-α*, *TRAF3*, *NF-κB*, *IRF1*, *IRF7* and others, were enriched in the Toll-like signaling pathway. Therefore, we analyzed the Toll-like signaling pathway, one of the main pathways of the host innate immune system [37], by GSEA and found that the Toll-like signaling pathway was significantly down-regulated overall at 24 h and 48 h. We verified the immune gene expression levels of this pathway by qPCR and found that *chERV3* induced significant (*p* < 0.05) downregulation of *IL-6* (24 h, 48 h), *IL-1β* (24 h, 48 h), *IRF7* (24 h, 48 h), *IFN-β* (24, 48 h), *IFN-α* (24, 48 h), *NF-κB* (24 h), and *TRF6* (24 h) expression; at 48 h, *chERV3* induced downregulation of *NF-κB* and *TRF6* expression, albeit not significantly (*p* > 0.05). The Toll-like receptor signaling pathway detects viral dsRNA molecules and activates two types of responses: the MyD88-dependent pathway, which induces inflammatory cytokine production, and the MyD88-independent pathway, which induces type I interferon (*IFN-α* and *IFN-β*) production [38]. Both pathways involve the activation of NF-κB and IRF factors, which regulate the expression of immune genes.

Key genes in the Toll-like signaling pathway, such as *NF-κB*, *IRF7*, and interferon genes, were down-regulated by *chERV3*. The inflammatory factors *IL-6* and *IL-1β* were also down-regulated by *chERV3*. We speculate that *chERV3* may inhibit the host immune response by blocking the Toll-like receptor signaling pathway and reducing the expression of inflammatory and antiviral factors. Nearly every cell in an organism needs *NF-κB* to operate, and *NF-κB* primarily interacts with TLRs, *TRAF6*, and *IL-1* genes [39]. Each member of the NF-κB family, including *p65* (RelA), *p50* (*NF-κB1*), *p52* (*NF-κB2*), RelB, and c-Rel, can combine with another member to form a dimer, which is the main component of the NF-κB pathway. Foreign pathogen invasion is recognized by cellular receptors after a series of signal transduction events, activating the IκB kinase complex (IKK), in turn inducing release of NF-κB dimers, and finally activating the NF-κB pathway [40], which induces inflammatory responses and tumor formation and promotes the release of inflammatory factors and tumor necrosis factors [41,42]. In chickens, eight IRF genes have been discovered, among which *IRF3/IRF7* are linked to innate immunity against viruses [43], and *IRF2*, *4*, and *7* promote both upregulation and downregulation of IFN expression [44]. *IRF7* is a protein that is only weakly phosphorylated under physiological conditions, but when a foreign pathogen invades the cell, *IRF7* is phosphorylated and translocated to the nucleus, where it binds to interferon-stimulated response elements to stimulate type I interferon pathway activation [45,46,47]. The results of GO enrichment analysis also showed that *chERV3* induced significant downregulation of DEGs enriched in NF-κB and type I interferon response processes. We hypothesized that *chERV3* may inhibit the type I interferon pathway and the NF-κB pathway by downregulating the Toll-like receptor signaling pathway, which may allow *chERV3* to evade the host innate immune response and persist in the genome. However, the mechanism by which *chERV3* affects the expression of Toll-like signaling pathway components is still unknown.

We also found that *chERV3* up-regulated many genes involved in the ECM receptor integration pathway. This pathway mediates the interactions between cells and the extracellular matrix (ECM), which is a network of proteins and carbohydrates that surrounds cells. One of the proteins in this pathway is THBS1, which can bind to MMPs, CD36, and CD47 [48]. These interactions can affect various signaling pathways, such as TGF-β signaling pathway and NF-κB signaling pathway [49,50,51]. CD36 is a membrane glycoprotein that is involved in lipid metabolism and immune tolerance. CD36 expression is increased in many types of cancer, such as acute myeloid leukemia, breast cancer, colorectal cancer, and gastric cancer [52,53]. Our analysis revealed significant upregulation of THBS1 and CD36 expression in the ECM receptor integration pathway. We hypothesized that *chERV3* may downregulate the host innate immune response and inhibit the NF-κB pathway by inducing CD36 expression, which may also allow *chERV3* to evade phagocytosis by macrophages. It is likely that *chERV3* inhibits the NF-κB pathway by inducing CD36 expression to suppress the innate immune response in chickens.

In summary, *chERV3* is able to suppress components of the host innate immune response, and we hypothesized that to achieve immune escape, *chERV3* may disrupt critical immune pathways such as the NF-κB and type I interferon pathways by blocking the Toll-like receptor signaling pathway. However, the mechanism by which *chERV3* affects the Toll-like receptor signaling pathway remains unclear.

## 5. Conclusions

In this study, we constructed a full-length reverse cloning plasmid (puc57-chERV3) in vitro. We used RNA-seq analysis to investigate how *chERV3* influences the host immune system 24 h and 48 h post transfection. The results showed that at 24 h and 48 h, *chERV3* prokaryotic expression induced significant downregulation of overall expression of Toll-like receptor signaling pathway components; in addition, GO enrichment analysis showed that *chERV3* induced downregulation of DEGs significantly enriched in NF-κB and type I interferon processes. We further screened for downregulation of *NF-κB*, *IRF7,* and other key components of the type I interferon pathway. The aim of this study was to preliminarily determine the effect of *chERV3* on the host innate immune response and to provide a theoretical basis for breeding disease-resistant chickens.

## Figures and Tables

**Table 1:** Comparison of gene statistics.

Sample Name	Integrity	Raw Reads	Clean Reads	Clean Bases	Q20	Total Mapped
NC 24 h1	8.2	43,381,886	41,480,772	6.22 G	96.3	37,555,122 (90.54%)
NC 24 h2	8.0	41,475,106	39,662,516	5.95 G	96.37	35,908,741 (90.54%)
NC 24 h3	8.5	42,085,432	40,226,640	6.03 G	96.52	36,489,585 (90.71%)
*chERV3* 24 h1	8.8	39,783,054	38,171,282	5.73 G	96.45	34,848,123 (91.29%)
*chERV3* 24 h2	8.9	42,270,748	40,115,088	6.02 G	96.55	36,638,260 (91.33%)
*chERV3* 24 h3	8.8	42,704,808	41,109,940	6.17 G	96.53	37,783,087 (91.91%)
NC 48 h1	6.3	41,127,206	39,467,128	5.92 G	96.01	35,673,640 (90.39%)
NC 48 h2	6.3	39,345,034	37,659,002	5.65 G	96.56	34,450,841 (91.48%)
NC 48 h3	5.8	41,363,842	39,521,786	5.93 G	96.36	35,821,588 (90.64%)
*chERV3* 48 h1	6.3	44,852,156	42,866,404	6.43 G	96.03	37,141,238 (86.64%)
*chERV3* 48 h2	8.0	39,628,688	37,820,374	5.67 G	96.46	34,638,935 (91.59%)
*chERV3* 48 h3	7.8	43,693,872	42,250,526	6.34 G	96.65	38,766,627 (91.75%)

**Table 2:** Information on immune-related DEGs.

Gene ID	Gene Name	24 h FPKM	log_2_(Foldchange)	48 h FPKM	log_2_(Foldchange)
ENSGALG00010006602	*GAS6*	1369.68/80.27	4.10	1286.21/210.06	2.61
ENSGALG00010017131	*TLR3*	1901.21/822.77	1.21		
ENSGALG00010006155	*TNFRSF19*	279.56/106.45	1.39		
ENSGALG00010001897	*TLR7*	112.44/42.96	1.39		
ENSGALG00010008392	*CD36*	41.77/5.97	2.81		
ENSGALG00010021550	*MAP2K3*	1603.22/6419.37	−2.00	1526.74/3871.01	−1.34
ENSGALG00010004987	*MAP3K8*	147.50/1357.06	−3.20	79.68/358.75	−2.16
ENSGALG00010029295	*SOCS3*	722.92/2295.62	−1.67	535.77/1592.99	−1.57
ENSGALG00010023847	*PIK3AP1*	182.21/834.92	−2.19	139.13/1993.38	−3.84
ENSGALG00010022134	*CH25H*	130.51/1500.54	−3.52	80.69/489.64	−2.61

## Data Availability

The data presented in this study are available on request from the corresponding author.

## Supplementary Materials

The following supporting information can be downloaded at: https://www.mdpi.com/article/10.3390/ani13172720/s1, Supplementary File S1:The primer information of full length *chERV3*, File S2:The primer information of qPCR, File S3: The SP value statistics, File S4: The FPKM value of *chERV3*, File S5: The information of DEGs induced by *chERV3*, File S6: GO terms and the information of DEGs involved in these GO terms, File S7:KEGG pathways and the information of DEGs involved in these pathways, Table S1: Analysis results of qPCR.
