# Supplementary figures and images for: Transcriptome Sequencing Reveals That Intact Expression of the Chicken Endogenous Retrovirus chERV3 In Vitro Can Possibly Block the Key Innate Immune Pathway
Source: Animals (Basel). 2023 Aug 26;13(17):2720. doi: 10.3390/ani13172720 (PMC10486640; doi:10.3390/ani13172720)

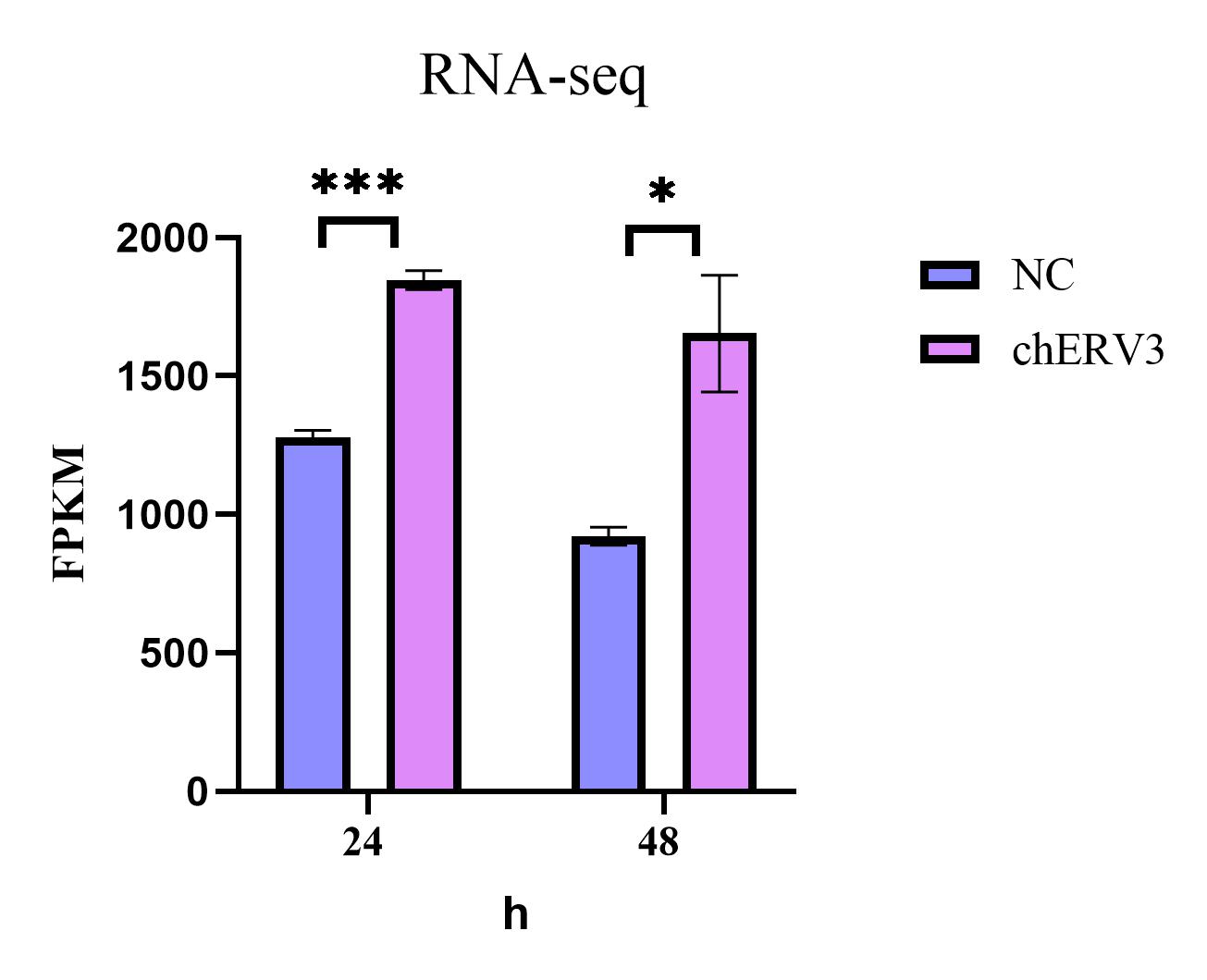

Supplement: Supplementary file 1 [file animals-13-02720-s001.zip › Supplentary File S4/chERV3 FPKM.jpg]
